# Low‐level laser therapy for skin rejuvenation: A safe and effective solution baked by data and visual evidence

**DOI:** 10.1111/jocd.16404

**Published:** 2024-05-30

**Authors:** Kawthar Shurrab, Juliana Nabil Alzghayar

**Affiliations:** ^1^ Higher Institute for Laser Research and Applications, Damascus University Damascus Syria

**Keywords:** low‐level laser therapy (LLLT), Melasma, rejuvenation, skin, wrinkles

## Abstract

**Background:**

Skin aging and photoaging is a process that may appear at a relatively early age, causing an aesthetic problem. Common signs of skin aging include wrinkles, dyspigmentation, and decreased elasticity.

**Aim:**

Aim of this article is to study the effectiveness and safety of low‐level laser therapy (LLLT) for skin rejuvenation.

**Methods:**

Thirty Syrian female patients aged 25–50 participated in this study and were diagnosed with moderate to severe melasma and wrinkles. The patients were divided into two groups and received LLLT with a wavelength of 660 nm emitting a continuous wave. The power density and dose used were 15.6 mW/cm^2^, and 3 J/cm^2^ respectively, and the laser effective area was 32 cm^2^. The evaluation was done before, during, and after 12 treatment sessions, based on photographs, in addition to the modified Melasma Area Severity Index (MASI), Pinch test, and Fitzpatrick's classification of facial wrinkling at baseline.

**Results:**

Comparing before, and after treatment, and between the two groups, revealed a significant improvement in skin rejuvenation, with a statistical significance (*p* < 0.05). Additional outcome measures included assessments of patient satisfaction scores, and no adverse effects or re‐pigmentation were reported.

**Conclusions:**

Our results suggest that LLLT may be a useful and safe therapeutic option in treating melasma, skin elasticity, and wrinkle improvement, which we advised to be integrated into treatment, and follow‐up programs in cosmetics and dermatology.

## 1 INTRODUCTION

Skin aging is affected by both temporal and environmental factors, but the most influential cause responsible for accelerating the skin aging process is photodamage resulting from excessive exposure to ultraviolet (UV) radiation.^1^

Low‐level light therapy (LLLT) is a fast‐growing technology, and a promising option to treat a multitude of conditions that require stimulation of healing, relief of pain and inflammation, and restoration of function.^2^ It is also considered a novel nonthermal and non‐ablative tool for skin rejuvenation. Which has been proven effective in improving several cosmetic conditions such as skin laxity and wrinkles.^3^ The skin responds well to red and near‐infrared (NIR) wavelengths. Where the laser photons are absorbed by mitochondrial chromophores in cells. This leads to an increase in adenosine triphosphate (ATP) nitric oxide release and improves blood flow and reactive oxygen species. Hence stem cells can be stimulated allowing increased tissue repair and healing.^4^, ^5^

Over the past few years, non‐ablative laser therapies, referred to as photorejuvenation, have gained popularity as an aesthetic treatment for fine wrinkles, photodamaged skin, and scars.^6^ LLLT involves applying low‐level energy or power densities of red and NIR light to cells or tissue. it is called “low‐level” because the energy employed is significantly lower compared to other laser treatments like ablation, cutting, and thermally coagulating tissue. Recently, it has been that medical treatment using this method has been found to work through a different mechanism, stimulating or inhibiting a variety of cellular processes.^7^, ^8^ The first signs of skin aging sometimes begin to appear in the late 20s, when wrinkles, dyspigmentation, loss of elasticity, and telangiectasia occur. At the histologic and molecular level, there is a decrease in the amount of collagen, fragmentation of collagen fibers, elastotic degeneration of elastic fibers, upregulation of matrix metalloproteinases (MMPs), especially MMP‐1 and MMP‐2.^9^

There are studies conducted by Abergel et al, and Yu et al,^10^, ^11^ showed that an increase in the production of pro‐collagen, collagen, and basic fibroblast growth factors (bFGF) after exposure to low‐level laser therapy (LLLT) in vitro and in vivo animal models. Schindl et al, and Ben‐Dov et al,^12^, ^13^ emphasized the role of low‐intensity laser irradiation on microcirculation in the skin with diabetic patients and affects satellite cell proliferation and differentiation in vitro. Bhat J et al,^14^ reported the efficacy of low‐intensity Omnilux Revive LED lamps in skin rejuvenation. Russell BA et al,^15^ determined the efficacy of combination LED light therapy (633 nm and 830 nm) in facial skin rejuvenation. Barolet D et al,^16^ highlighted that a pulsed 660 nm LED light source affects the regulation of skin collagen metabolism. Neil S Sadick et al,^17^, ^18^, ^19^, ^20^, ^21^ demonstrated the success of using a portable LED device to treat skin aging using wavelength 830 nm, and 633 nm.

Previous studies have agreed on the impact of low‐power lasers and LED devices on the skin and demonstrating their results in treatment. One of the most prominent differences between previous studies was the variety of wavelengths used, application time, treatment duration.

While most of the previous studies used LED low intensity light and two wavelengths, our study emphasized the role of a single wavelength laser diode 660 nm emitting a continuous wave. The power density and dose used were 15.6 mW/cm^2^, and 3 J/cm^2^ respectively, and the laser effective area was 32cm^2^. In addition to determining the appropriate session time and exposure time for safe and effective treatment using LLLT on skin rejuvenation.

## 2 CLINICAL ASSESSMENT

A series of color facial photographs were taken prior to, during, and following the sessions, after 2 weeks, 4 weeks, and 6 weeks. The outcomes were evaluated at the commencement, during, and upon completion of the treatment.

To evaluate the melasma we used a modified Melasma Area and Severity Index (MASI) scoring system.^22^, ^23^ The MASI score is computed through a subjective evaluation of two variables: the area of involvement (A), and the darkness (D). The forehead (f), right malar region (rm), left malar region (lm), and chin (c) correspond to 30%, 30%, 30%, and 10% of the total face, respectively. The total score ranges from 0 to 24.

For the skin elasticity was assessed, according to the pinch test scale, which is as follows^24^:

1‐ We face the palm of the hand downwards in a relaxed manner, then pinch the skin on the back of the hand and hold for 5 s.

2‐ We release the skin and count the time it takes until it becomes completely flat.

We then record the score as follows:

1–2 s (individuals under 30); 3–4 s (individuals who are 30–44); 5–9 s (individuals who are 45–50); 10–15 s (individuals who are 60); 35–55 s (individuals who are 70+).

To assess skin wrinkles, we used the Fitzpatrick's Classification of Facial Wrinkling, which is as follows^25^, ^26^, ^27^:

Class I, 1–3: fine wrinkles to mild (fine textural changes with subtly accentuated skin lines).

Class II, 4–6: fine to moderate depth wrinkles, moderate number of lines moderate.

Class III, 7–9: fine to deep wrinkles, numerous lines, with or without redundant skin Severe.

## 3 METHODS

Before treatment, informed consent was taken from all patients and they were asked about their concerns and expectations regarding the treatment to avoid unrealistic expectations. They were educated about the dangers of excessive exposure to sunlight.

Patients were instructed to always apply a broad‐spectrum sunscreen with a sun protection factor of more than 30 throughout the treatment period, not to use any chemicals or cosmetics products on the skin during the treatment period, and to have the skin clean.

The patients did not take any medications that caused or worsened pigmentation and they did not use any type of chemical peels for a year, and there was no allergy. A red diode laser was applied with a wavelength of 660 nm (Konftec, Red Laser 660 nm /250 ~ 500 mW, Model No. emlas‐650, Laser Shower), emitting a continuous wave. The power density and dose used were 15.6 mW/cm^2^, and 3 J/cm^2^ respectively, and the laser effective area was 32 cm^2^. The treatment began on March 15, 2023, until May 15, 2023. No topical anesthetics or medications were administered before, during, or after the treatment. The face was divided into six areas (forehead, around the eyes, cheeks, and around the mouth), and each area was irradiated for 6 min to treat melasma and pigmentation, and 4 min to treat facial wrinkles with one pass. The patients did not feel any discomfort, redness, or any side effects. Receiving treatment stopped after 12 sessions to be evaluated and follow‐up was done during treatment, and monthly for 6 months posttreatment. There were no negative effects occurred, and no repigmentation was reported. They were divided into two groups:

Group A: two sessions per week for 6 weeks.

Group B: three sessions per week for 4 weeks, making a total of 12 sessions in each group.

A questionnaire was given to patients at the end of treatment to assess their satisfaction with the degree of improvement, and it was rated as mild (<25% improvement), moderate (25%–<50%), good (50%–<75%), and very good (>75% improvement).

The SPSS (version 24) was used to check, enter, and analyze data. Data were represented as mean ± SD for quantitative variables.

The paired *t*‐test, and independent *t*‐test were used to assess the results. *p* < 0.05 was considered statistically significant.

## 4 RESULTS AND DISCUSSION

This study included 30 female patients, their ages ranged from 26 to 50 years with a mean of 39.4 ± 6.7. According to Fitzpatrick's skin type; seven patients had skin phototype II (23.3%), 20 patients with phototype III (66.6%), and three patients with phototype IV (10%). The duration of pigmentation and melasma ranged from 3 to 15 years with a mean of 5.64 ± 4.82. Family history was detected in 16 patients (53.3%). The effect of sun exposure was found in 30 patients (100%). in five patients (16.6%) the appearance of pigmentations occurred related to pregnancy. The wrinkles were due to intense exposure to sunlight and age. 56.7% had previous treatment with chemical peels and whitening products.

Demographic and clinical data for the study groups are illustrated in Table 1 and Table 2.

**TABLE 1:** Demographic data of the study groups.

Variable	(*n* = 30)
Age (year)	
Range	26–50
Mean ± SD	39.4 ± 6.7
Sex	
Female	100%
Male	0%
Duration of pigmentation (year)	
Range	3–15 years
Mean ± SD	5.64 ± 4.82
Skin type	
II	23.3%
III	66.6%
IV	10%
Previous treatment (chemical peels)	
No	13 patients (43.4%)
Yes	17 patients (56.7%)

**TABLE| 2:** Significant difference in MASI score, pinch test, and wrinkles before and after treatment within Group A.

Group A	Number of patients	Mean	Std. deviation	*df*	*t*	Significant difference
MASI scale	15	Pre 13.7 After 4.8	2.4 1.9	14	9.3	< 0.05
Skin elasticity	15	Pre 6.4 After 2.6	3.1 1.2	14	7.3	< 0.05
Facial wrinkles	10	Pre 5.3 After 1.6	0.8 0.5	9	24.2	< 0.05

The outcomes showed a significant improvement after using LLLT for skin rejuvenation in both groups after conducting 12 sessions for each patient. Where the difference was clear in the decrease of pigmentation and melasma during the treatment stages. As shown in Figure 1, (A) a 39‐year‐old woman from Group A, showed noticeable improvement in the appearance of the skin during the treatment stages with LLLT, after 2 weeks, after 4 weeks, and after 6 weeks at the end of 12 sessions. While Figure 1, (B) depicts, a 35 years old woman from Group B, where the decrease in the pigmentation and melasma also noticeably appeared.

**FIGURE 1:**
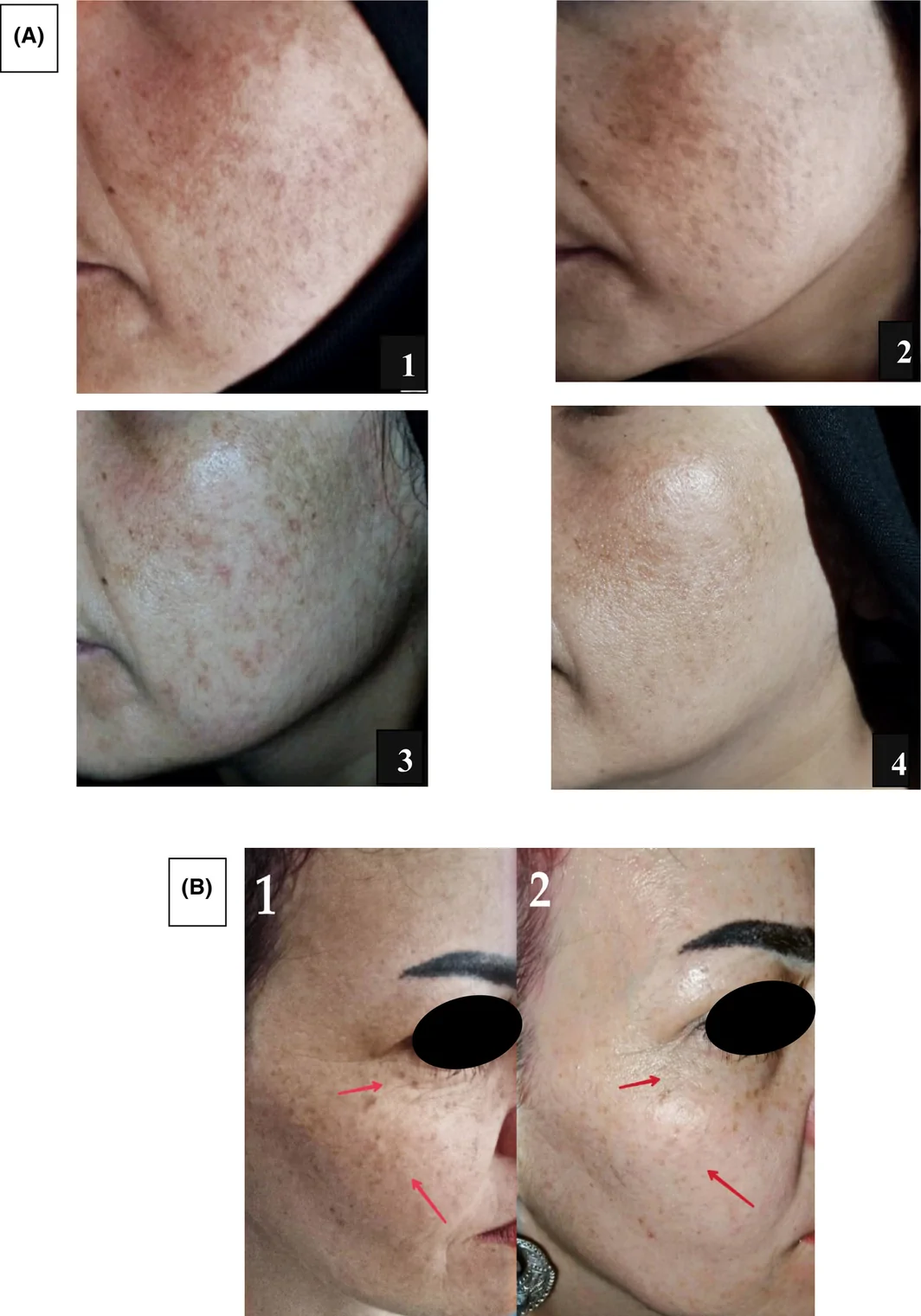
A decrease in pigmentation and melasma in a patient from a) group A, No. 1 is before treatment with LLLT, No.2 is after 2 weeks, No.3 is after 4 weeks and No.4 is after 6 weeks at the end of 12 sessions (two sessions per week). b) and Group B, No. 1 is before treatment with LLLT, and No.2 is after 4 weeks at the end of 12 sessions (Three sessions per week).

There was also a significant improvement in wrinkles around the eyes, as displayed in Figure 2, (A) 46‐year‐old woman from group 1, showed noticeable improvement in the appearance of the eye wrinkles during the treatment stages with lllt, after 2 weeks, after 4 weeks, and after 6 weeks at the end of 12 sessions. while Figure 2, (B) illustrates, a 42 years old woman from Group B, where the wrinkles are clearly improved as well.

**FIGURE 2:**
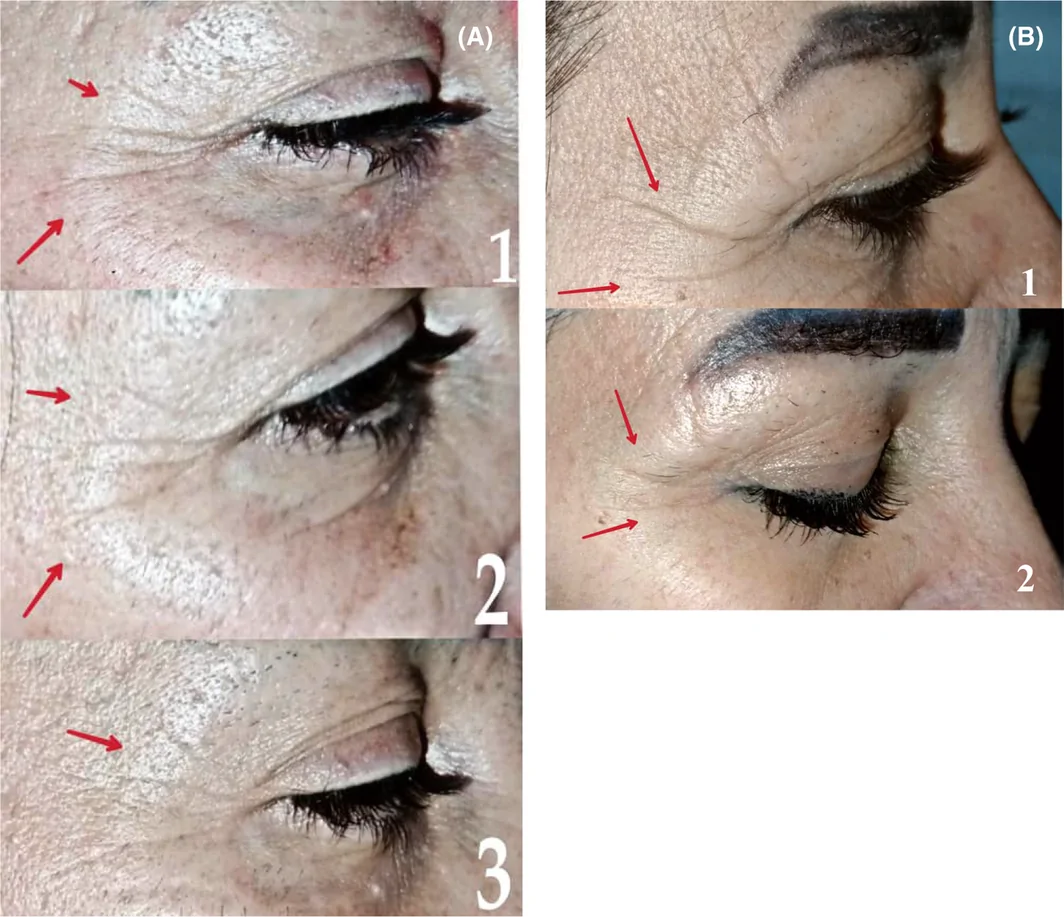
A decrease in eye Wrinkles in a patient from (A) Group A, No. 1 is before treatment with LLLT, No.2 is after 4 weeks, No.3 is after 6 weeks at the end of 12 sessions (two sessions per week). (B) and Group B, No. 1 is before treatment with LLLT, and No.2 is after 4 weeks at the end of 12 sessions (Three sessions per week).

The results we obtained confirm the effectiveness and safety of LLLT in the study groups. Both in Group A, and Group B, by using a continuous wave diode laser in red with a wavelength of 660 nm, the power density and dose used were 15.6 mW/cm^2^, and 3 J/cm^2^ respectively, and the laser effective area was 32 cm^2^. The 12 sessions of LLLT are considered sufficient to produce extreme improvement in skin rejuvenation, whether two or three sessions each week. To our knowledge, this is the first study that emphasizes the role of LLLT while specifying the number of sessions and the required dose to achieve that.

According to patient satisfaction; five patients (16.6%) reported their improvement as mild, 18 patients (60%) reported their improvement as good, and seven patients (23.3%) as very good.

In fact, there was a big enhancement in skin rejuvenation for all patients who participated in this study, but the patient evaluation was based on their personal expectations.

### 4.1 Data analysis

All the parameters were assessed using SPSS software version 24. A Paired *t*‐test was performed to find the statistical difference within the groups before and after LLLT, to compare the MASI score, improved skin elasticity, and facial wrinkles, and independent t‐test was adopted to find statistical difference between the two groups. There was a significant difference in Group A, MASI score (related to reduced pigmentation and melasma) before (M = 13.7, *SD* = 2.4) and posttreatment (M = 4.8, *SD* = 1.9); *t* (14) = 19.3, *p* < 0.05.

There was also a significant difference in the Pinch test about skin elasticity pre‐ (M = 6.4, *SD* = 3.1) and posttreatment (M = 2.6, *SD* = 1.2); *t* (14) = 7.3, *p* < 0.05.

As well as a significant difference in facial wrinkle improvement before (M = 5.3, *SD* = 0.8) and posttreatment (M = 1.6, *SD* = 0.5); *t* (9) = 24.2, *p* < 0.05. As represented in Figure 3, and Table 2.

**FIGURE 3:**
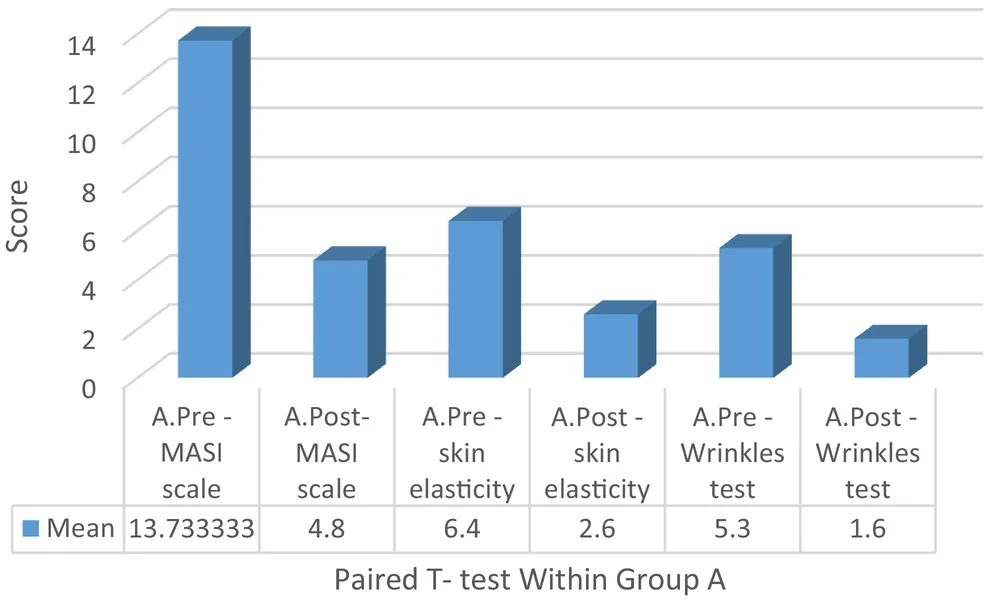
Graphical representation of significant difference in MASI score, pinch test, and wrinkles within Group A.

As for Group B, there was a significant difference in MASI score (related to reduced pigmentation and melasma) before (M = 14.2, *SD* = 2.6) and posttreatment (M = 5.7, SD = 1.5); *t* (14) = 17.8, *p* < 0.05.

And a significant difference in the Pinch test about skin elasticity pre‐ (M = 6.4, SD = 3.1) and posttreatment (M = 3.6, *SD* = 1.8); *t* (14) = 7.1, *p* < 0.05.

As well as a significant difference in facial wrinkle improvement before (M = 5.6, SD = 0.8) and posttreatment (M = 1.8, *SD* = 0.6); *t* (9) = 15.2, *p* < 0.05. As listed in Figure 4, and Table 3.

**FIGURE 4:**
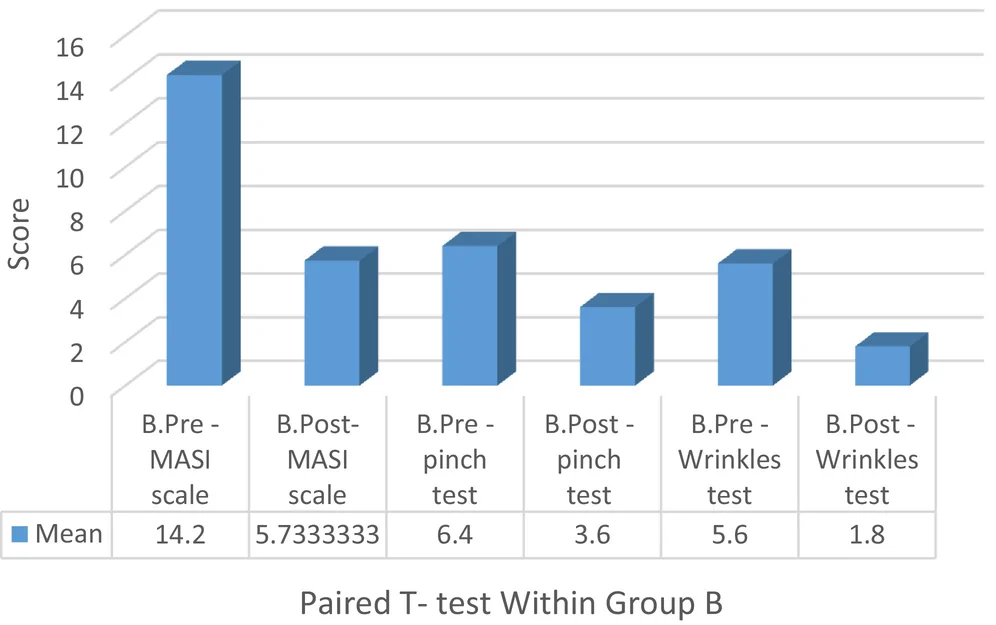
Graphical representation of significant difference in MASI score, pinch test, and wrinkles within Group B.

**TABLE| 3:** Significant difference in MASI score, pinch test, and wrinkles within Group B.

Group B	Number of patients	Mean	Std. deviation	*df*	*t*	Significant difference
MASI scale	15	14.2 5.7	2.6 1.5	14	17.8	< 0.05
Skin elasticity	15	6.4 3.6	3.1 1.8	14	17.1	< 0.05
Facial wrinkles	10	5.6 1.8	0.8 0.6	9	15.2	< 0.05

An Independent Samples *T*‐test was performed to compare the reduced MASI score, improved skin elasticity, and wrinkles between group A (two sessions per week), and Group B (three sessions per week).

The outcomes show that there were no significant differences in MASI score (related to reduced pigmentation and melasma) between Group A (M = 5.3, *SD* = 2.2), and Group B (M = 4.5, *SD* = 1.6); *t* (25) = 1.029, *p* = 0.312.

And there were no significant differences in pinch test score (skin elasticity) between Group A (M = 2.5, *SD* = 1.2), and Group B (M = 2.7, *SD* = 1.4); *t* (28) = 0.282, *p* = 0.780.

As for Fitzpatrick's Classification (wrinkles improvement) also there were no significant differences between Group A (M = 2.1, *SD* = 0.73), and Group B (M = 2.3, *SD* = 0.67); *t* (18) = 0.632, *p* = 0.535. as represented in Figure 5, and Table 4.

**FIGURE 5:**
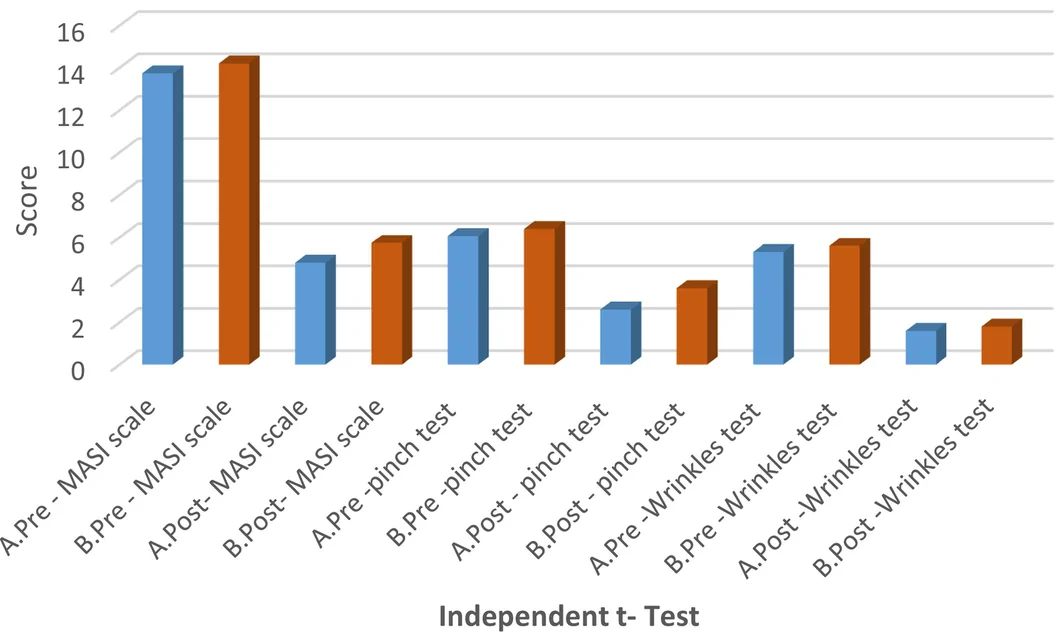
Comparative independent samples test between Group A, and B.

**TABLE| 4:** Independent SAMPLES TEST for MASI score, pinch test, and wrinkles between Group A, and B.

Variables	Test	Group A mean	Group B mean	*df*	*t* Value	*p* < 0.05
MASI Score	Pre test	14.7	14.2	28	0.6	N0
	Post test	5.3	4.5	27	1	N0
Skin elasticity	Pre test	6.4	6.6	28	0.194	N0
	Post test	2.5	2.6	28	0.288	N0
Facial wrinkles	Pre test	5.5	5.2	18	0.774	N0
	Post test	2.2	2.2	18	0.772	N0

According to patient satisfaction; 20 patients (66.6%) reported their improvement as very good, six patients (20%) depicted their improvement as good, and four patients (13.3%) as moderate.

There were no significant statistical differences between the first group A, in which the treatment continued for 6 weeks, and the second group B, in which the treatment continued for 4 weeks, but in general we noticed that the rate of improvement on the skin was slightly better in the second group in terms of the speed of the appearance of results.

There are also statistically significant differences in the improvement of the appearance and elasticity of the skin between the cases of the research sample according to their different ages, and these statistically significant differences are in favor of the cases from the age group (30–50) because the average degrees of improvement for them is more and greater than the average degrees of improvement for the cases from the age group (29 years and younger).

The second group, which received 12 sessions within 4 weeks, excelled in shortening the duration of application of the treatment and thus obtaining results slightly faster than the first group, because of the intensification of the sessions specified with a short time interval between one session and the next.

## 5 CONCLUSION

Our study revealed that the use of a low‐energy laser with a wavelength of 660 nm within 12 sessions represents a safe and effective treatment for pigmentation and improves the appearance of the skin in general. There is also a statistically significant difference in the pre and post‐treatment with a *p* <0.05. We recommended a longer treatment duration and more sessions for best results. In addition, we suggest that further studies be performed at different power densities, and wavelengths and compared to those used in our study.

## FUNDING INFORMATION

None.

## CONFLICT OF INTEREST STATEMENT

No conflict of interests.

## ETHICS STATEMENT

The authors have followed all of the ethics publishing terms.

## CONSENT

Written informed consent was obtained from the patient to publish this report in accordance with the journal's patient consent policy.

## Data Availability

All data are included in the manuscript.
